# Exploring Eco-Sustainability in Functionally Unconventional Magnetic Field Sensors

**DOI:** 10.1021/acs.nanolett.5c05155

**Published:** 2026-01-06

**Authors:** Rui Xu, Denys Makarov

**Affiliations:** Institute of Ion Beam Physics and Materials Research, 28414 Helmholtz-Zentrum Dresden-Rossendorf e.V., Bautzner Landstrasse 400, Dresden 01328, Germany

**Keywords:** magnetic field sensors, printing electronics, transparent electronics, flexible electronics, sustainability

## Abstract

Magnetic field sensors are indispensable components in modern electronics owing to their reliable, contactless operation. Over the last decades, the rapid advancement of emerging technologies (e.g., wearable devices, transparent electronics, virtual and augmented reality, soft robotics, and the Internet of Things) has not only fueled the expanding demand for magnetic field sensors but also imposed increasingly stringent requirements on their performance and functionality. However, conventional fabrication processes, predominantly based on thin-film techniques, often entail energy-intensive procedures and excessive material waste, generating significant environmental impacts. Furthermore, the intrinsic rigidity and opacity of traditional sensors hinder their seamless integration into next-generation platforms. In response, the research community has undertaken extensive efforts to reconcile unconventional functionality with environmental sustainability. This review highlights recent advances in this direction, focusing on strategies that endow magnetic field sensors with mechanical flexibility and optical transparency while simultaneously addressing sustainability challenges throughout their entire life cycle.

## Graphical abstract

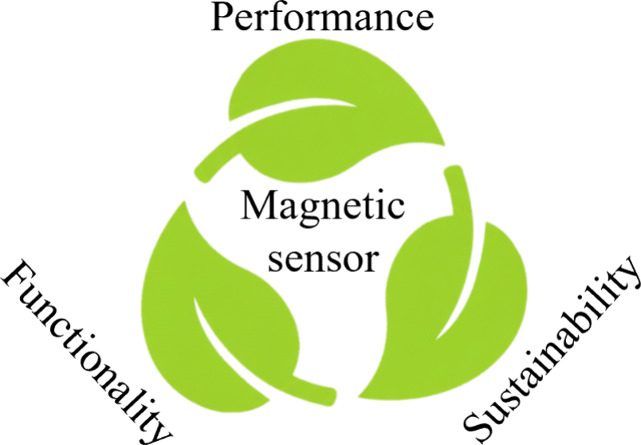

## Introduction

Magnetic field sensors, renowned for their response to magnetic fields and touchless operational modes, have been extensively employed in monitoring diverse motion states including orientation, rotation, and motion (Figure 1). Contemporary magnetic field sensing technologies are primarily based on diverse magnetoresistance (MR) and Hall-effect-based mechanisms (for a detailed comparison of different sensing mechanisms, please refer to several comprehensive review papers).^1–6^ Owing to their robustness and reliability, magnetic field sensors have become indispensable components across navigation, environmental monitoring, industrial automation, consumer electronics, and medical devices. In recent years, the rapid expansion of emerging technologies, such as wearable electronics, smart health monitoring, virtual/augmented reality systems, soft robotics, and Internet-of-Things (IoT) platforms, has dramatically broadened the application landscape of magnetic field sensors, driving fast market growth while simultaneously raising stringent requirements for performance and functionality.

**Figure 1:**
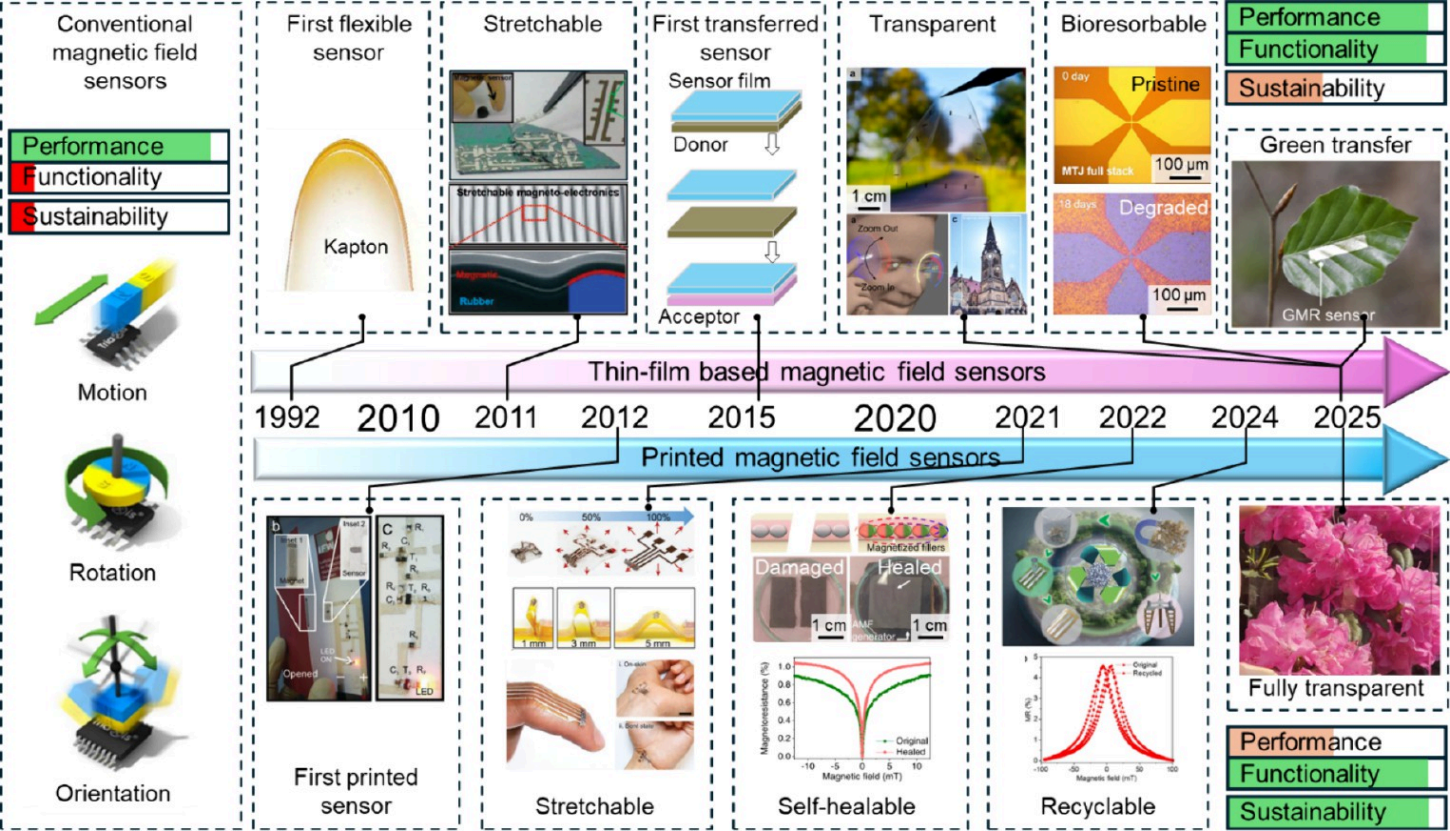
Development roadmap of unconventional magnetic field sensors. The evolution from conventional rigid and opaque sensors toward mechanically conformable (flexible and stretchable) and optically transparent designs is driven by two key technological pathways: thin-film fabrication combined with transfer techniques (top panels) and additive printing methodologies (bottom panels). Reproduced with permission.^12^ Copyright 2011, American Chemical Society. Reproduced with permission.^13^ Copyright 2025, Springer Nature Ltd. with a Creative Commons CC-BY license. Reproduced with permission.^21^ Copyright 2025, John Wiley and Sons. Reproduced with permission.^15^ Copyright 2025, John Wiley and Sons with a Creative Commons CC-BY license. Reproduced with permission.^16^ Copyright 2012, John Wiley and Sons. Reproduced with permission.^17^ Copyright 2021, John Wiley and Sons with a Creative Commons CC-BY license. Reproduced with permission.^19^ Copyright 2022, Springer Nature with a Creative Commons CC-BY license. Reproduced with permission.^20^ Copyright 2024, Royal Society of Chemistry with a Creative Commons CC-BY license. Reproduced with permission.^18^ Copyright 2025, American Chemical Society with a Creative Commons CC-BY license.

This surge in demand is reflected in global production statistics: in 2022, the global shipment of magnetic field sensors exceeded 10 billion units with forecasts predicting an increase to more than one trillion units by 2030.^7,8^ Conventional fabrication of magnetic field sensors typically relies on mature thin-film technologies, typically involving high-vacuum deposition of magnetic materials combined with lithographic patterning and etching. While these approaches ensure high performance and reproducibility, they are inherently associated with complex processing, high energy consumption, and limited material utilization. Furthermore, magnetic compositions commonly employed such as Ni and Co are classified as hazardous under the Globally Harmonized System of Classification and Labeling of Chemicals (GHS). Improper handling or disposal may pose significant toxicological threats to humans and ecosystems. Likewise, silicon wafers used as the dominant substrates demand energy-intensive manufacturing, rely on large volumes of ultrapure water and corrosive chemicals, and generate hazardous waste that is difficult to treat. Collectively, these factors raise substantial concerns about the environmental sustainability of conventional magnetic field sensors.

From an application standpoint, the rigidity and opacity of silicon substrates impose additional constraints on integration into emerging technologies. Their poor mechanical adaptability hinders stable operation in flexible, skin-conformal, or wearable devices, where repeated deformation such as bending and stretching is unavoidable.^9^ The light-blocking nature of both silicon and thin-film magnetic layers further limits their use in systems where optical information transmission is crucial, such as smart windows, transparent displays, and wearables.^10^ The lack of such critical attributes presents a major barrier to the deployment of traditional sensors in next-generation multifunctional platforms. These limitations collectively underscore the pressing need for device engineering strategies that not only introduce unconventional functionalities but also address sustainability concerns.

In this context, the scientific community has initiated a series of explorations aimed at reconciling functionality with sustainability. Figure 1 outlines the pioneering development of various unconventional magnetic field sensors, highlighting the key innovations that have driven this technological transition. Early studies explored ultrasmooth polymeric substrates as alternatives to silicon, offering reduced environmental impact along with enhanced mechanical flexibility and optical transparency.^11–13^ To further extend device integration across substrates of different materials and geometries, transfer-printing techniques were subsequently developed.^14,15^ Afterward, printing-based fabrication methods have attracted particular attention, owing to their straightforward low-energy and material-efficient nature.^16–18^ More recently, research has progressed toward magnetic field sensors with self-healing, recyclability, and bioresorbable capabilities, thereby mitigating the urgent issue of electronic waste (e-waste).^19–21^ Together, these efforts signify a paradigm shift toward greener and adaptable sensor technologies with the potential to fundamentally transform the magnetic field sensor industry.

Nevertheless, research in this direction is still in a relatively early stage. Fully realizing the vision of magnetic field sensors that combine high performance, environmental compatibility, and unconventional properties will require interdisciplinary collaboration across materials science, device physics, fabrication engineering, and end-of-life management. Previous reviews have primarily focused on specific aspects of these emerging devices, including novel fabrication strategies (e.g., solution-processable printing technologies, thin-film based transfer printing methods),^22–24^ novel physical attributes (e.g., mechanical flexibility/stretchability, optical transparency, biocompatibility, biodegradability),^3,25–30^ and diverse application domains (spanning biomedicine, healthcare, robotics, human–machine interaction, and wearable electronics).^31–36^ However, a perspective on the sustainable development of these functionally unconventional sensors remains lacking. The present review aims to summarize recent advances in magnetic field sensor technologies from a sustainability perspective. Particular emphasis is placed on identifying critical opportunities and persistent challenges that must be addressed to further mitigate the environmental footprint of the magnetoelectronics industry and to guide future research toward more eco-efficient technological pathways. The discussion is organized following the sensor lifecycle encompassing fabrication, utilization, and disposal.

### i Ultrasmooth Polymeric Substrates as Replacements for Silicon Wafers in Thin-Film Sensors

Compared with silicon wafers, polymer-based substrates can be fabricated through more energy-efficient and resource-saving processes. These characteristics render them not only easier to produce but also more compatible with the overarching paradigm of green and environmentally responsible manufacturing. A broad range of polymeric materials have been explored as hosts for magnetic materials such as polyimide (PI),^37^ polyethylene terephthalate (PET),^38^ polyimide (PVDF),^39^ polyether ether ketone (PEEK),^40^ polyester,^41^ and polydimethylsiloxane (PDMS).^42^ On these substrates, diverse types of thin-film magnetic field sensors have been successfully fabricated. For example, Parkin and co-workers were the first to demonstrate giant magnetoresistance (GMR) sensors by depositing Co/Cu multilayers onto Kapton substrates.^11^ Subsequently, Wang et al. reported anisotropic magnetoresistance (AMR) sensors prepared by sputtering 30 nm permalloy (Py) films onto PET foils, with a sensitivity as high as 42 T^–1^.^43^ Melzer et al. showed that bismuth-film Hall sensors exhibit a sensitivity of approximately −2.3 V (AT)^−1^, which remains essentially independent of the choice of polymeric substrates, whether PEEK or PI.^40^ Furthermore, Co/Al_2_O_3_/NiFe magnetic tunnel junctions sputtered onto both Kapton and PI foils delivered tunneling magnetoresistance (TMR) ratios of ∼12%.^44^ These findings confirm that polymeric foils are capable of supporting complex multilayer spintronic architectures, thereby offering an effective route for the development of eco-friendly magnetoelectronic devices.

The substitution of traditional silicon substrates with flexible alternatives represents not only a step toward sustainable fabrication technology but also a functional breakthrough in magnetic field sensors. For instance, AMR sensors sputtered onto PET foils exhibit sufficient flexibility to conformally attach to curved surfaces such as the human fingers or wrists (Figure 2a,b).^43^ Further reduction in substrate thickness enables stable device operation under extreme bending radii as small as 150 μm.^45^ Similar trends were observed in flexible GMR sensors (Figure 2c–e)^41,46–49^ and Hall sensors (Figure 2f–j),^50–52^ highlighting the strong correlation between substrate conformability and device resilience. Beyond bendability, functional diversification has been realized by employing stretchable polymers. When substrates are prestrained prior to magnetic material deposition and subsequently released, spontaneous micro- or nanoscale surface patterning occurs, imparting stretchability to the devices and thus allowing for seamless integration with dynamic interfaces (Figure 2k,l).^12,42,53–55^ Building on these advances, Lugoda et al. integrated, for the first time, flexible GMR sensors into the core of textile braids, thereby creating magnetoreceptive smart textiles with remarkable mechanical robustness and washability for everyday wear (Figure 2m,n).^56^ When combined with transparent substrates such as Mylar foils, mesh-like patterning imparts optical transparency to magnetic field sensors, with transparency exceeding 85% (Figure 2o–q).^13^ These studies confirm polymeric substrates as promising enablers for magnetic field sensors with unconventional properties, thereby enabling their seamless integration into a wide range of application scenarios, such as wearables,^56–58^ smart health monitoring,^49^ transparent electronic systems,^13^ and soft robotics.^32,59,60^ Notably, achieving ultrahigh transparency demands markedly low surface coverage of magnetic materials in combination with highly precise patterning, which in turn results in considerable material wastage and a substantially more intricate fabrication process. Similarly, attaining extreme mechanical compliance often requires the incorporation of specialized polymers as substrates or protective layers, the fabrication of which may rely on substantial energy consumption, harsh processing conditions, and the use of environmentally hazardous chemicals. These factors highlight a trade-off between mechanical and optical functionality and environmental sustainability, underscoring the need for careful material selection, process optimization, and lifecycle considerations in the design of flexible transparent magnetoelectronic devices.

**Figure 2:**
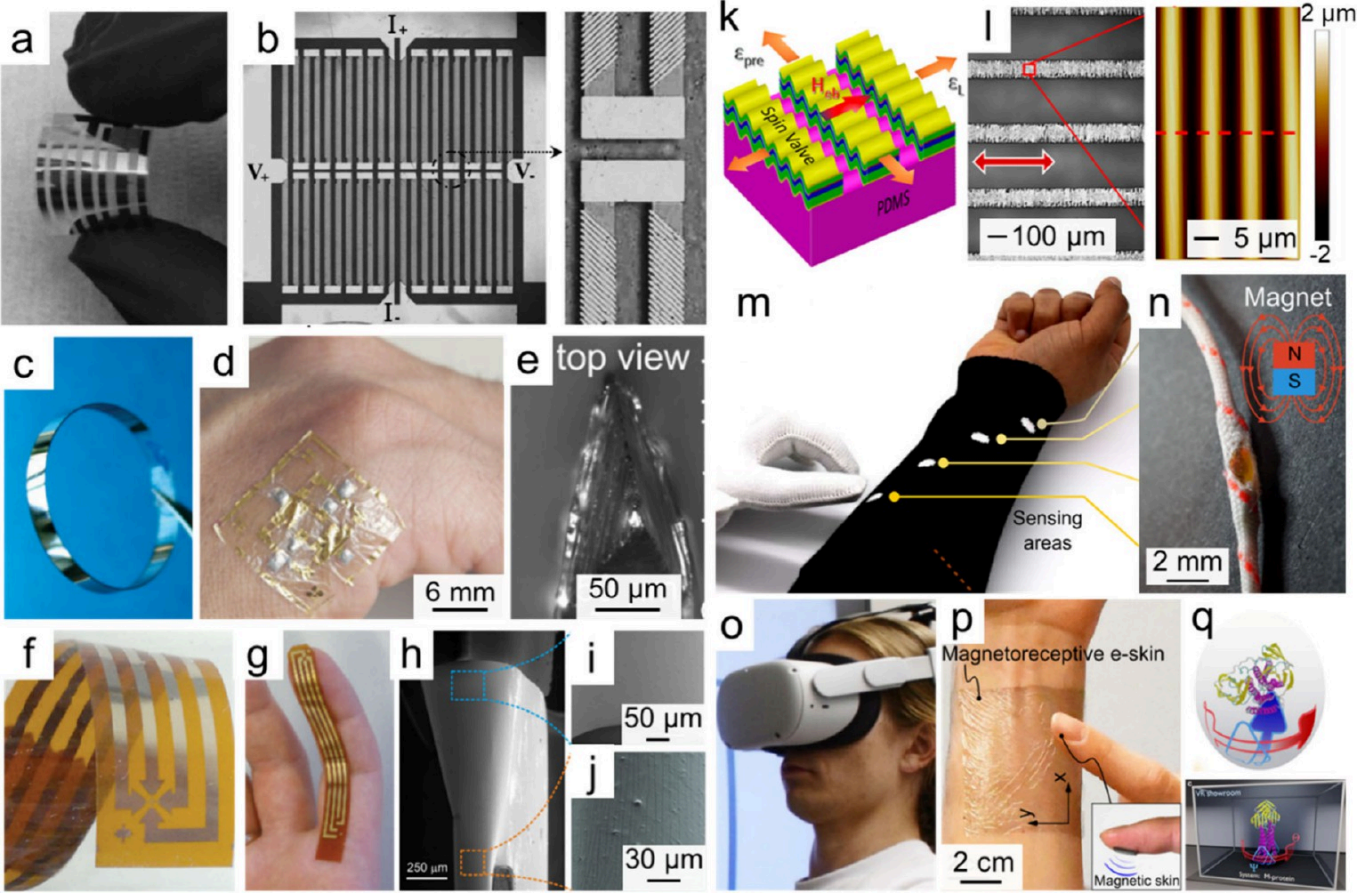
Magnetic field sensors on flexible substrates. a,b) Py-based AMR sensors fabricated on PET substrates: a) optical photography; b) microscope image of sensing elements. Reproduced with permission.^43^ Copyright 2016, John Wiley and Sons. c) GMR Co/Cu multilayers on polyester substrates. Reproduced with permission.^39^ Copyright 2008, John Wiley and Sons. d,e) Conformable GMR elements on ultrathin foils, demonstrating adaptability to d) human skin and e) a doctor blade. Reproduced with permission.^48^ Copyright 2018, The American Association for the Advancement of Science with a Creative Commons CC-BY license. f,g) Bismuth film-based Hall sensors: f) device structure and g) conformal attachment on a human finger. Reproduced with permission.^40^ Copyright 2015, John Wiley and Sons with a Creative Commons CC-BY license. h,i) Bent planar Hall sensor: h) overall morphology under bending radii of approximately 100–300 μm; i,j) zoomed-in views corresponding to curvature radii of 290 and 110 μm, respectively. Reproduced with permission.^50^ Copyright 2019, Springer Nature Ltd. with a Creative Commons CC-BY license. k,l) Stretchable GMR sensor: k) schematic illustration and l) optical image with surface morphology characterization of the GMR layer on a wrinkled elastomeric substrate. Reproduced with permission.^54^ Copyright 2025, American Chemical Society. m,n) Interactive textile-based magnetic sensing system: m) knitted sleeve integrated with four overbraided GMR sensors and a magnetic glove; n) amplified image revealing the internal overbraided sensor structure. Reproduced with permission.^56^ Copyright 2025, Springer Nature Ltd. with a Creative Commons CC-BY license. o–q) Transparent and flexible GMR mesh applied as magnetoreceptor for virtual interaction: o) a user operating in a virtual reality (VR) environment; p) arm-mounted GMR mesh allows q) accurate spatial control of virtual entities. Reproduced with permission.^13^ Copyright 2025, Springer Nature Ltd. with a Creative Commons CC-BY license.

Despite the encouraging advances, the selection of suitable substrates remains severely constrained by the interfacial sensitivity of magnetic thin films. Multilayered GMR structures, for example, are governed by delicate spin-dependent coupling and scattering processes, which are highly susceptible to even subtle interfacial imperfections. Unlike silicon wafers, which exhibit atomically smooth surfaces (<0.5 nm roughness), polymer substrates typically exhibit higher surface roughness values (root-mean-square, 1–10 nm), resulting in elevated magnetic noise and degraded sensing performance. This limitation is more pronounced in TMR devices, where surface irregularities amplify tunneling current nonuniformities and even trigger electrical short circuits.^18,44^ One common strategy to mitigate this issue is the introduction of buffer layers. For instance, Chen et al. demonstrated that the introduction of a photoresist layer can effectively reduce the surface roughness of plastic substrates to below 1 nm, comparable to that of thermally oxidized silicon. This surface improvement resulted in an enhancement of GMR ratios by as much as 200%.^41^ Building upon this approach, Wang et al. further developed anisotropic magnetoresistance (AMR) sensors exhibiting a remarkably enhanced sensitivity of 42 T^–1^, which is comparable to that of reference devices on silicon.^43^ In addition to surface roughness, other intrinsic challenges stem from the limited thermal robustness of polymers. Compared to Si wafers, which can withstand temperatures as high as 1000 °C, most polymeric substrates tolerate substantially lower thermal loads (typically, <400 °C). High-temperature annealing essential for optimizing magnetic thin films often induce substrate deformation, interfacial stress, or chemical reactions, which compromise device integrity and long-term stability.^61–63^ Thus, while polymer substrates hold considerable promise for multifunctional magnetic field sensors, their integration remains hindered by fundamental interfacial incompatibilities.

In summary, polymeric substrates present a dual-edged opportunity: they provide sustainability benefits and unconventional device functionalities, yet their surface and stability limitations impose critical trade-offs. Addressing these challenges requires the development of substrate platforms that combine ultralow surface roughness with enhanced thermal/chemical resistance, while retaining environmental compatibility.

### ii Transfer Methods for Unlocking Substrate Limitations of Thin-Film Sensors

To broaden the range of applicable substrates to include materials with improved environmental benignity and recyclability while maintaining high device performance, transfer techniques have been developed. The central concept of this approach is to first fabricate high-performance sensing elements on donor substrates under well-controlled conditions and subsequently relocate them onto target substrates while retaining their structural and functional integrity. Rogers and his co-workers significantly advanced the development of transfer printing techniques for diverse micro- and nanoscale patterns composed of a wide range of functional materials (including metals, semiconductors, oxides, and organics).^64–66^ In general, three representative strategies have been established for the transfer of thin-film elements. 1) Leveraging the weak van der Waals interactions in two-dimensional (2D) layered systems, magnetic films can be mechanically exfoliated from donor substrates with minimal damage.^67–69^ 2) The intrinsically low adhesion strength between SiO_2_ and certain metallic layers (e.g., Au, Ni) can also be utilized to facilitate the controlled detachment of magnetic films.^70,71^ 3) Alternatively, sacrificial layers can be deliberately introduced beneath magnetic films; their selective dissolution in suitable solvents or etchants enables efficient release of the active layers and subsequent integration with target substrates.^72–75^

Based on these strategies, thin-film magnetic field sensors have been integrated onto different substrates. For example, Loong et al. reported transferable TMR sensors, in which magnetic tunnel junctions were initially fabricated on silicon substrates to allow high-temperature annealing for improved crystalline and interfacial properties (Figure 3a). Following dry etching of the sacrificial silicon layer (Figure 3b), the sensing patterns were relocated onto flexible PET substrates (Figure 3c), yielding high-performance flexible TMR devices.^76^ Similarly, Melzer and colleagues demonstrated the transfer of GMR sensors onto PDMS substrates. The strain-released substrates generated wrinkled surface patterns, imparting excellent mechanical conformability to the sensors and ensuring comfortable wearability.^14^ However, their approach relied on harsh processes such as dry/wet etching, plasma treatment, or mechanical bonding at elevated temperatures, which not only caused partial performance degradation but also increased environmental impact. To address these limitations, we proposed a water-assisted transfer method that exploits the high surface tension of water to facilitate stress-free placement (Figure 3f,g), thereby preserving the pristine magnetoresistance characteristics without any measurable degradation.^15^ This technique enables reliable transfers onto a variety of curved substrates with excellent structural integrity (Figure 3h).^15^ Importantly, the proposed approach circumvents the use of harsh treatments and toxic chemicals, thereby avoiding potential damage to receiver substrates, as evidenced by successful application of a broad range of materials, e.g., plastics, ceramics, textiles, wood, and even biological surfaces (Figure 3i,j). It can be anticipated that transferred sensors exhibit weaker interfacial adhesion between the sensing elements and the underlying substrates compared with directly deposited counterparts. Such reduced adhesion may compromise long-term operational stability, particularly under continuous mechanical deformation in flexible applications. To mitigate these limitations, strategies such as thermal treatment, plasma/chemical surface activation, or mechanical pressing can play critical roles in strengthening interfacial bonding and enhancing device reliability.^14,15,76^

**Figure 3:**
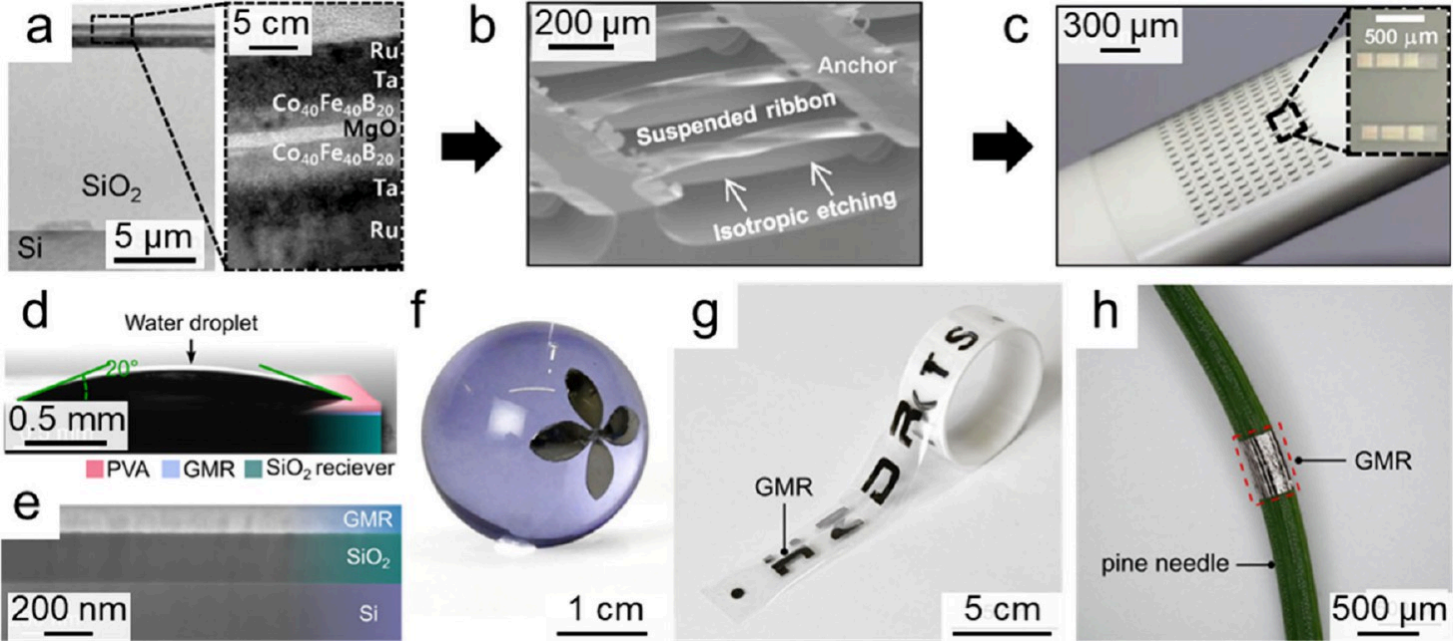
Magnetic field sensors transferred onto diverse substrates. a–c) Flexible TMR sensors transferred onto PET substrates. a) Magnetic tunnel junction film deposited on a surface-oxidized Si substrate. b) Selective etching of Si to release the junction structure. c) Transfer of the released sensor onto a PET substrate. Reproduced with permission.^76^ Copyright 2016, John Wiley and Sons. d–h) Water-enabled eco-friendly transfer of GMR sensors onto a wide spectrum of substrates. d) Demonstration of the hydrophilic surface of a PVA-coated GMR film, beneficial for controlled transfer. e) Interface evaluation of transferred GMR layers, revealing intimate adhesion to receiver substrates. f–h) Examples of GMR films transferred onto f) a glass sphere, g) PVA-based adhesive tape, and h) a pine needle. Reproduced with permission.^15^ Copyright 2025, John Wiley and Sons with a Creative Commons CC-BY license.

In principle, transfer methodologies provide a versatile route to seamlessly integrate magnetic field sensors onto substrates with arbitrary compositions and geometries, thereby expanding their applicability beyond silicon wafers and ultrasmooth polymers toward more sustainable platforms. Such advances hold great promise for wide deployment of magnetic sensing technologies, contributing to the vision of ubiquitous connectivity in a greener future society. Nevertheless, compared with conventional thin-film electronics, transferrable electronics generally exhibit lower production efficiency, limited by their multistep fabrication and transfer processes including device fabrication on a temporary substrate, precise alignment, and transfer printing. Additionally, it should be emphasized that the core fabrication still heavily relies on vacuum-based deposition of magnetic layers and precise micro/nanoscale patterning. These process requirements continue to pose significant barriers to fully aligning transfer-based approaches with the principles of sustainable manufacturing. To be fair, neither direct deposition on polymeric substrates nor transfer-based fabrication routes alone can comprehensively address the full range of sustainability challenges associated with magnetic field sensors. However, each of these approaches mitigates specific limitations inherent to conventional silicon-based technologies, thereby representing meaningful advances toward more sustainable device platforms.

### iii Additive Printing Techniques for Energy- and Material-Efficient Sensor Fabrication

In contrast to conventional thin-film fabrication routes, additive printing techniques offer a particularly sustainable pathway for magnetic field sensor manufacturing. Their inherently straightforward, layer-by-layer workflow minimizes material wastage, reduces energy consumption, and avoids reliance on expensive vacuum systems or sophisticated lithographic facilities. Beyond process sustainability, printing also relaxes substrate requirements, enabling sensor integration on low-cost supports such as paper, ceramics, textiles, polymers, etc.^16,17,77^ This combination of ecological advantages and design freedom has stimulated broad interest in printed magnetic field sensors.

The versatility of this approach is reflected in the wide range of printable devices reported to date. For instance, Karnaushenko et al. pioneered the development of printable magnetic field sensors using inks composed of multistacked Py/Cu GMR flakes dispersed within poly­(methyl methacrylate) (PMMA) matrices (Figure 4a,b).^16^ Subsequent refinements by the same group demonstrated that a rational tailoring of polymeric binders could markedly boost device performance.^77^ Notably, the introduction of polyepichlorohydrin (PECH) binders elevated MR response from an initial 7% to an impressive 37%. In parallel, inks formulated with Co/Cu multisegmented nanowires as functional fillers enabled GMR sensors with MR ratios up to 14% (Figure 4c,d).^78^ The scope of printable magnetic devices extends beyond GMR. AMR sensors have been realized using inks incorporating Py flakes^79^ and microparticles.^19^ Leveraging Bi microparticles as the active medium, our group has demonstrated nonsaturating large magnetoresistance (LMR) sensors with record-high MR ratios approaching 146% at room temperature (Figure 4e,f).^80^ Furthermore, researchers successfully fabricated Hall sensors employing hierarchical nickel nanowires or graphene (Figure 4g,h).^81,82^ Collectively, these studies underscore the exceptional material and structural adaptability of printing technologies, capable of accommodating fillers ranging from Ni, Co, and Py to Bi, and morphologies spanning microparticles, nanowires, and flakes, thus establishing a versatile platform for next-generation magnetic field sensors.

**Figure 4:**
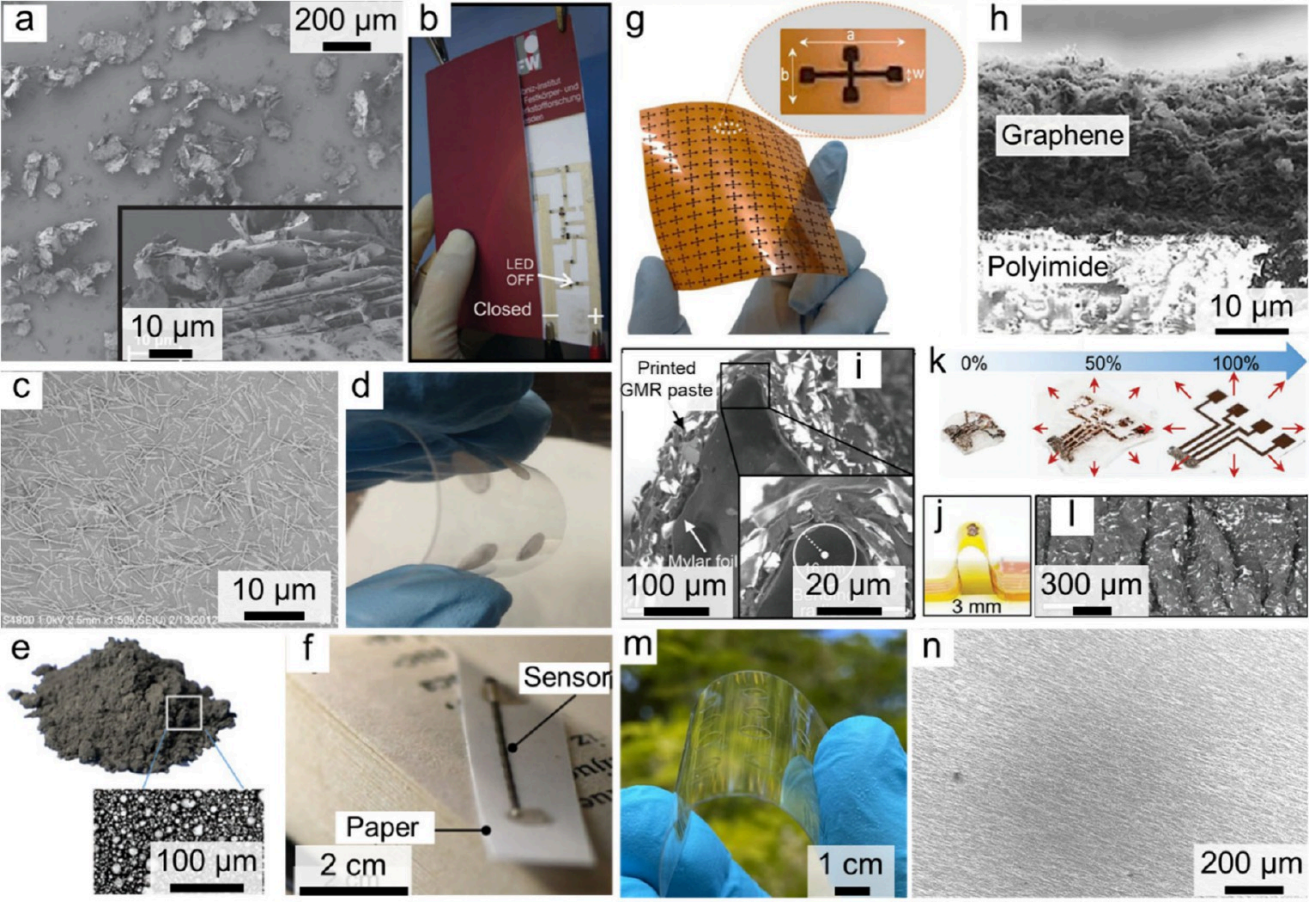
Overview of printed magnetic field sensors fabricated with different functional materials and structural designs. a,b) GMR sensors printed using Co/Cu multilayer flakes as fillers: a) SEM image of Co/Cu GMR flakes; b) printed device on postcard paper serving as part of a hybrid electronic circuit. Reproduced with permission.^16^ Copyright 2012, John Wiley and Sons. c,d) GMR sensors incorporating Py/Cu multisegment nanowires: c) SEM image of the nanowires; d) printed nanowire network on polymer substrates. Reproduced with permission.^78^ Copyright 2013, Elsevier. e,f) LMR sensors printed with bismuth microparticles: e) optical and SEM images of bismuth fillers; f) printed sensor on paper. Reproduced with permission.^80^ Copyright 2022, John Wiley and Sons with a Creative Commons CC-BY license. g,h) Graphene-based printed Hall sensors: g) optical image of printed sensor arrays; h) cross-sectional SEM image showing graphene on polyimide. Reproduced with permission.^81^ Copyright 2021, Springer Nature Ltd. with a Creative Commons CC-BY license. i–l) Mechanically adaptive GMR sensors: i) SEM and j) optical images of bent sensors; k) optical images under 0%, 50%, 100% strain conditions; l) SEM image after strain release. Reproduced with permission.^17^ Copyright 2021, John Wiley and Sons with a Creative Commons CC-BY license. m,n) Transparent magnetic field sensors printed with Py nanowires: m) optical image of arrayed sensors printed on flexible substrates; n) directionally aligned Py nanowire network. Reproduced with permission.^18^ Copyright 2025, American Chemical Society, with a Creative Commons CC-BY license.

The high design freedom of printing techniques leads to magnetic field sensors with unique functional attributes. By optimizing binders and substrates, Karnaushenko et al. demonstrated GMR sensors with remarkable mechanical flexibility, bearing bending to radii as small as 12 mm without discernible performance degradation.^77^ Further advances in conformability were reported by Ha et al., who utilized strain-release Mylar foils to realize ultradeformable GMR devices with bending radii down to 16 μm and stretchability up to 100% (Figure 4i–l).^17^ Remarkably, this level of mechanical deformability rivals that of state-of-the-art thin-film and transferred devices, which typically exhibit bending radii on the order of tens of micrometer scales while offering stretchability elevated by tens to hundreds of percents.^13,14,53^ Likewise, optical transparency has been pursued through the use of nanowire-shaped fillers: Cox et al. achieved semitransparent GMR sensors by employing Py/Cu multisegmented nanowires, where the high aspect ratio facilitated mesh-like architectures with reduced optical scattering.^78^ More recently, directional alignment of nanowires under tilted magnetic fields during printing was proposed, yielding sensors with optical transparency up to ∼85% (Figure 4m,n).^18^ In contrast, achieving comparable optical transparency typically requires limiting the surface coverage of thin-film magnetic field sensors to approximately 10%,^13^ a requirement that not only results in significant material wastage but also imposes stringent demands on high-precision patterning techniques. Therefore, additive printing technologies provide a more sustainable and versatile route for imparting transparency and compliance to magnetic field sensors to enable new functionalities.

Despite these advances, printed magnetic field sensors continue to face significant performance limitations compared with their thin-film counterparts. For example, the maximum MR ratios of printed AMR sensors sharply reduced from 1.9% to 0.34%.^79^ A similar trend was observed in printed GMR sensors, where the MR ratio dropped from ∼20% to 6%^16^ and from ∼40% to <30%.^83^ Such performance degradation may arise from multiple factors. First, the percolative nature of filler networks introduces unavoidable contact resistances, diminishing electrical conductivity. Second, the random orientation of fillers disrupts the alignment of magnetic domains and spin-dependent conduction pathways, leading to lower sensitivity and device reproducibility. Third, due to the limited thermal stability of binder polymers (typically <400 °C), thermal annealing and related post-treatments at high temperatures, which are essential for improving filler crystallinity and interfacial properties, are often impractical. As a result, printed sensors are less competitive for applications requiring ultrahigh precision. Instead, their intrinsic advantages (e.g., low cost, scalable fabrication, mechanical adaptability, and environmental compatibility) render them more appealing to applications where moderate performance is sufficient, such as smart home systems, environmental monitoring, and Internet-of-Things (IoT) devices. Another important limitation lies in the resolution constraints of current printing techniques. Unlike lithography, which readily achieves nanoscale feature definition, most printing methods are restricted to microscale patterning, impeding the realization of ultracompact or multifunctional integrated systems. Overcoming this bottleneck by improving printing resolution, while simultaneously closing the performance gap with thin-film devices, thus constitutes a key research priority for the future development of printed MR sensors.

In fact, the realization of fully sustainable magnetic field sensors still remains a considerable challenge. In general, the environmental impact of printed magnetic field sensors is strongly governed by the chemical nature of the solvents, surfactants, magnetic particles, and polymeric binders employed during ink formulation and device fabrication. For example, organic solvents may give rise to concerns associated with volatile organic compound emissions, flammability, and ecological toxicity, thereby compromising the overall sustainability of printed magnetic field sensor technologies. Surfactants commonly used to stabilize filler dispersions typically exhibit limited biocompatibility and biodegradability and may accumulate in ecosystems, imposing additional environmental burdens. Magnetic particles introduce further considerations. For instance, the extensive use of Co- and Ni-based materials poses potential health risks to humans as well as substantial ecological impact. Their nanoscale dimensions may complicate end-of-life recovery and recyclability. Likewise, many commonly used polymeric binders exhibit poor biodegradability and often require energy-intensive processing or harsh chemical treatments for recycling, limiting opportunities for circular reuse. Consequently, the adoption of greener solvent systems (e.g., water or bioderived solvents), biodegradable polymer binders (e.g., starch, cellulose, chitosan), and low-toxicity magnetic nanomaterials (such as Fe- or iron-oxide-based formulations) will be critical for further advancing the sustainability of printed magnetic field sensor technologies.

### iv In-Service Self-Healing and Responsible End-of-Life Management

The escalating challenge of electronic waste (e-waste) has emerged as a pressing global issue. As indispensable components integrated into a vast array of electronic platforms, magnetic field sensors are expected to experience continuous growth, which, in turn, amplifies their contribution to the e-waste stream. Although the unique material compositions and structural designs of the above magnetic field sensors enable new functionalities, they also introduce potential reliability challenges, especially when introduced into IoT nodes, wearables, or soft robotics. For example, when integrated into IoT nodes, harsh outdoor environments exacerbate aging mechanisms. In wearables, continuous body contact, sweat, and biofluids introduce additional chemical and mechanical stresses that challenge the longevity. In soft robotics, persistent dynamic deformation can accelerate mechanical fatigue and interface failure. In this context, self-healing and responsible end-of-life management have been proposed to extend device lifetimes and reduce e-waste generation, thereby alleviating the environmental footprint of magnetic field sensors.

Embedding self-healing capability provides a practical strategy to prolong service life, reduce replacement frequency, and slow the accumulation of e-waste. Typically, this is achieved by formulating printed composites with polymeric binders that exhibit intrinsic self-healing properties.^84^ Upon mechanical damage, dynamic covalent bonds, supramolecular interactions, or reversible physical cross-linking within the polymer matrix facilitate autonomous repair, restoring structural continuity. Simultaneously, viscoelastic recovery drives the redistribution of functional fillers toward damaged sites, enabling the partial reconstruction of percolation pathways. However, residual polymer layers between adjacent fillers can persist, impeding charge transport and compromising electrical performance and, thus, the reliability of the healing process. However, many of the conventional strategies to boost the self-healing functionality are difficult to translate into MR composites. For instance, increasing temperatures can promote binder mobility and thus provide stronger driving forces for filler migration; however, it also accelerates the surface oxidation of magnetic fillers, severely compromising their electrical and magnetic properties. To circumvent these limitations, we introduced an alternating magnetic field (AMF)-assisted self-healing strategy.^19^ By actively manipulating the orientation and mobility of magnetic fillers during the healing process, AMF treatment promotes dipole–dipole interactions and oscillatory motion, driving the formation of elongated filler chains and enhanced interfiller contacts (Figure 5a–d). This mechanism accelerates the re-establishment of conductive percolation networks, enabling efficient functional recovery (Figure 5e–g). Notably, the AMF-driven self-healing exhibits six key advantages: 1) complete recovery of device performance; 2) reliable repeatability across multiple cycles; 3) rapid restoration minimizing functional downtime; 4) operation at ambient temperatures, protecting temperature-sensitive components; 5) robustness against environmental variations such as humidity; and 6) autonomous recovery without the need for manual intervention. Collectively, these attributes underscore AMF-assisted healing as a viable approach to enhance the resilience and reliability of MR sensors in service.

**Figure 5:**
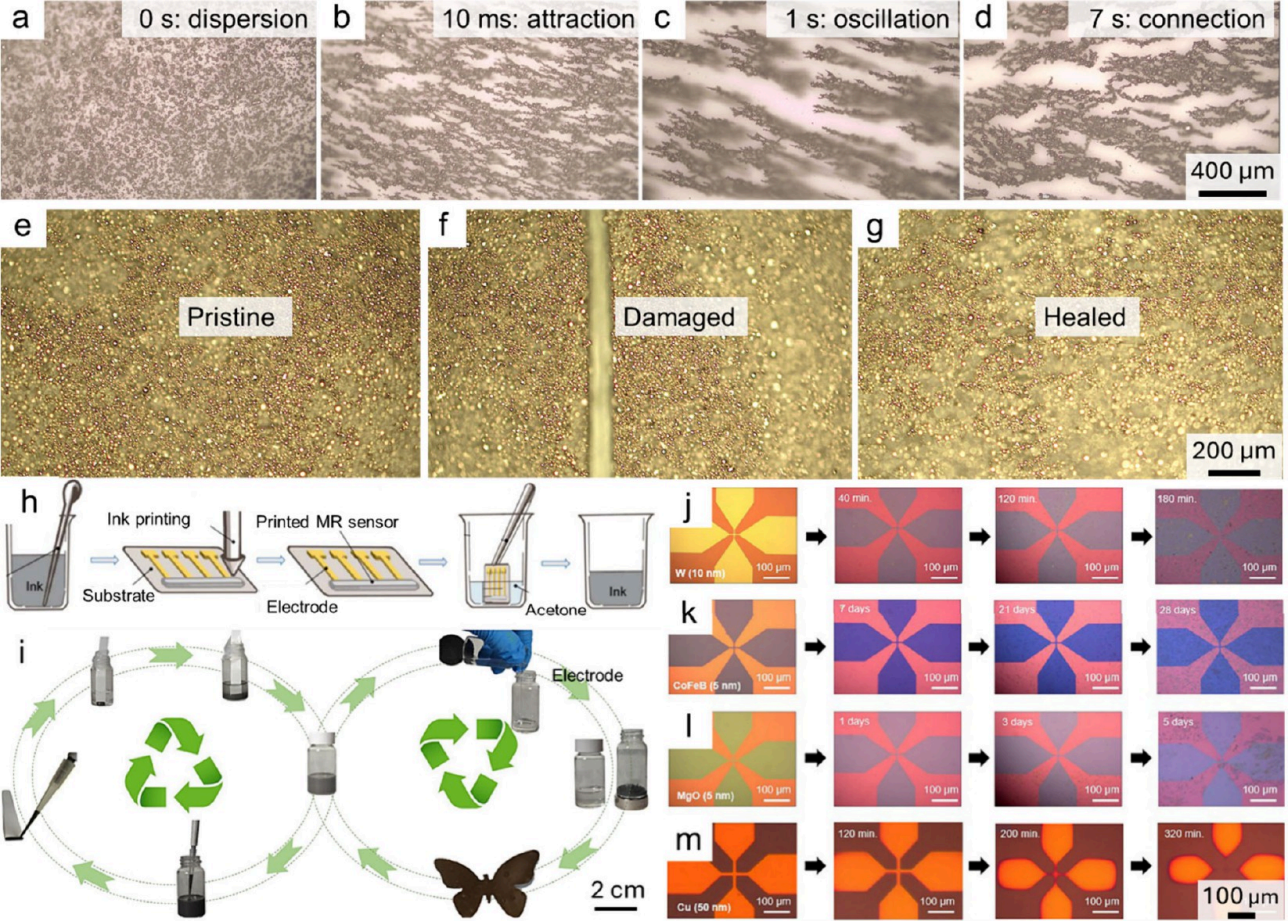
Self-healable, recyclable, and bioresorable magnetic field sensors. a–d) Real-time interaction of Py microparticles within printed inks under alternating magnetic fields, illustrating distinct filler behaviors: a) dispersion, b) attraction, c) oscillation, and d) connection. The values shown in the top-right corners represent the elapsed time after the application of magnetic fields. e–g) Self-healing process of the sensor assisted by alternating magnetic fields: e) pristine state, f) damaged with a scratch, and g) fully healed. Reproduced with permission.^19^ Copyright 2022, Springer Nature with a Creative Commons CC-BY license. h,i) Recyclable printed magnetic field sensors: h) schematic illustration of fabrication and recycling process, leveraging redissolvable polyepichlorohydrin (PECH) as binders, and (i) two recyclable approaches. The left cycle depicts the refabrication route, where discarded sensors are dissolved in solvent to disintegrate polymer binders and magnetic fillers, and the recovered ink is reused for printing new devices. The right cycle presents the material separation route in which the extracted polymers and magnetic particles are repurposed to construct a magnetic butterfly-shaped soft robot. Reproduced with permission.^20^ Copyright 2024, Royal Society of Chemistry with a Creative Commons CC-BY license. j–m) Time-lapse optical images capturing the stepwise degradation of MTJ constituent materials in phosphate-buffered saline (PBS) solutions at 37 °C, as indicated by Hall-bar patterns of j) 10 nm W film, k) 5 nm CoFeB, l) 5 nm MgO, and m) 50 nm Cu, revealing progressive dissolution of each layer over time. Reproduced with permission.^21^ Copyright 2025, John Wiley and Sons.

While self-healing extends operational lifetimes, recycling and biodegradation are equally critical for sustainable e-waste management. Improper disposal, particularly through landfilling, risks the release of toxic elements such as Ni and Co, leading to environmental contamination and adverse health outcomes. To address this issue, we employed PECH as the binder in printed MR sensors (Figure 5h).^20^ Upon immersion of discarded sensors in acetone, PECH readily dissolves, enabling the facile separation of the polymer matrix from the magnetic fillers (Figure 5i). Leveraging the intrinsic magnetic responsiveness of the fillers, they can be efficiently recollected using a simple external magnetic field, thereby minimizing the material loss (Figure 5i). This process establishes a controlled disassembly route in which both the polymer and magnetic components can be recycled as secondary raw materials for subsequent device fabrication. Such a closed-loop approach not only reduces the risk of environmental contamination but also promotes circular resource utilization. Recently, Kim et al. exploited the ultrathin nature of magnetic tunnel junction (MTJ) structures, successfully realizing the complete dissolution of TMR sensing components in phosphate-buffered saline (PBS) solutions at 37 °C (Figure 5j–m).^21^ This bioresorbable behavior under physiological conditions not only highlights the potential of TMR sensors for transient operation and secure data eradication in biomedical applications but also inspires future efforts toward magnetoelectronic systems that can autonomously degrade in natural environments.

In summary, the integration of in-service self-healing with end-of-life recycling delineates a promising technological framework for responsible sensor development. Together, these strategies extend device longevity, minimize replacement frequency, and provide sustainable end-of-life pathways, thereby mitigating the environmental burdens associated with the rapid expansion of sensor technologies.

## Perspective

Despite the remarkable progress, the pursuit of eco-sustainable magnetic field sensors with unconventional properties remains a highly intricate and multifaceted endeavor. A truly sustainable roadmap must adopt a holistic view that spans the entire sensor lifecycle, from material selection and fabrication to deployment and eventual disposal. Within this broader context, several key directions can be envisioned to guide future research and innovation.

1. Enhancing device performance. For sustainable sensors to gain competitiveness in practical applications, continued performance improvement remains paramount. In the case of printable devices, multiple strategies hold promise. Incorporating nanostructured fillers (such as nanowires or nanoflakes) can significantly expand interparticle contact areas, thereby facilitating the construction of more robust and conductive percolation networks. Complementarily, controlled mild post-treatments, for instance localized laser heating, may improve interfacial bonding without compromising the integrity of polymer binders. The application of external fields during printing represents another avenue, as electric, magnetic, or acoustic guidance can promote filler alignment, ensuring pathway continuity and improved carrier transport. Equally important are advances in ink formulation and process optimization, which can enhance the homogeneity of printed architectures, collectively leading to substantial gains in sensitivity and reliability. In addition, the inclusion of two-dimensional materials into magnetic sensing architectures potentially expands the design space for next-generation sensors. Their exceptional physical and electronic properties (such as atomic-level uniformity, tunable electrical property, and strong spin–orbit coupling) enable the realization of unconventional sensing mechanisms and device configurations.^85–87^

2. Artificial intelligence (AI) driven sensor intelligence. From a system-level perspective, AI-supported conditioning could empower magnetic field sensors to transcend traditional threshold-based operating paradigms and dynamically interpret complex magnetic interactions. By leveraging machine learning algorithms for adaptive signal processing and feature extraction, such systems can uncover latent spatiotemporal correlations embedded in fluctuating magnetic fields, enabling the discrimination of subtle variations in movement, rotation, or orientation patterns that would otherwise remain undetectable through conventional methods. This data-driven approach not only enhances the perceptual acuity of magnetic sensing platforms but also facilitates context-aware decision-making, allowing the system to infer user intent or environmental states with higher fidelity. Ultimately, the convergence of AI and magnetic sensing paves the way toward intelligent, self-optimizing, and responsive wearable or implantable systems capable of continuous adaptation to real-world dynamics.
3. Biocompatibility and material choices. Alongside technical performance, the ecological and biological compatibility of magnetic field sensors is a critical factor for their sustainable deployment. Fe, one of the most abundant and physiologically essential elements, offers a compelling basis for environmentally benign sensors. Yet, Fe-based devices often fall short in terms of sensitivity and magnetoelectric response, highlighting the need to balance biocompatibility with functional performance. Beyond magnetic materials, other device constituents, including substrates and polymeric binders, must also be carefully considered. Natural polymers such as cellulose, chitosan, gelatin, and silk fibroin provide attractive candidates, offering renewable origins, broad availability, and inherent flexibility, while simultaneously minimizing environmental and health risks.
4. Green recycling technologies. Conventional recycling approaches, dominated by high-temperature treatments and aggressive chemical processes, remain effective yet problematic, often generating secondary pollution. Future strategies should integrate sustainability considerations at the design stage, where careful material selection can simplify disassembly and resource recovery. The adoption of water-soluble binders or easily separable substrates, for example, may enable recycling through mild physical or chemical treatments, achieving a balance among efficiency, feasibility, and ecological responsibility.
5. Biodegradable pathways. Beyond technical recycling strategies, the systemic challenge of device collection continues to impede the development of circular electronics. Divergent regulatory frameworks, inconsistent infrastructure, and heterogeneous consumer practices hinder the effective channeling of discarded devices into formal recycling streams. In this regard, biodegradable magnetic field sensors present a particularly compelling vision. By decomposing into benign byproducts after use, such devices could seamlessly reintegrate into natural ecological cycles, circumventing the need for specialized retrieval or treatment and fundamentally reducing the burden of e-waste management.
6. Balance between sustainability and functionality. When assessed from a life-cycle perspective, the aforementioned magnetic field sensors exhibit notable environmental and sustainability advantages compared with conventional thin-film counterparts, albeit accompanied by trade-offs in performance and durability. For instance, printed sensors minimize material consumption and energy expenditure, whereas sensors directly sputtered onto polymeric substrates offer enhanced device performance. Transferred sensors expand the range of compatible unconventional substrates but require multistep fabrication processes that elevate energy and material demands. Bioresorbable sensors and recyclable sensors, respectively, mitigate electronic waste or enable material recovery at the end of life, but introduce constraints related to material stability, processing compatibility, and long-term device reliability. Collectively, these considerations underscore an inherent trade-off between environmental impact and device functionality, which must be carefully balanced in the future.
7. Emerging application scenarios. The unique combination of eco-friendliness and unique characteristics positions unconventional magnetic field sensors to unlock novel application domains. Intelligent packaging systems could leverage these sensors to dynamically monitor food quality and logistics conditions. Magnetoreceptive smart textiles not only provide a multifunctional interface enabling dynamic interaction with external magnetic environments but also function as an active safety alert system for users with magnetically sensitive implantable electronic devices. The proximity and orientation sensitivity of magnetic field sensors can equip soft robotics with advanced spatial awareness, enabling them to perceive and interact with their surroundings in a more autonomous and adaptive manner. In biomedicine, biocompatible and even implantable sensors may offer real-time physiological monitoring while ensuring safety and eventual biodegradation. Within environmental monitoring, large-scale deployment of transient sensors in natural ecosystems could provide distributed data acquisition, with the added advantage of natural decomposition into harmless residues. Collectively, such developments not only advance the vision of a “smart society” but also pave the way for a truly “green society”, where technology and sustainability are synergistically intertwined.
